# A global overview of healthcare workers’ turnover intention amid COVID-19 pandemic: a systematic review with future directions

**DOI:** 10.1186/s12960-022-00764-7

**Published:** 2022-09-24

**Authors:** Yuan-Sheng Ryan Poon, Yongxing Patrick Lin, Peter Griffiths, Keng Kwang Yong, Betsy Seah, Sok Ying Liaw

**Affiliations:** 1 Alice Lee Centre for Nursing Studies, Yong Loo Lin School of Medicine, National University of Singapore, Singapore, Singapore; 2 Nursing Service, Tan Tock Seng Hospital, Singapore, Singapore; 3 National Institute for Health and Care Research (NIHR) Applied Research Collaboration (Wessex), University of Southampton, Southampton, UK; 4 Group Nursing, National Healthcare Group, Singapore, Singapore

**Keywords:** COVID-19 pandemic, Healthcare workers, Turnover intention

## Abstract

**Background:**

Globally, the health workforce has long suffered from labour shortages. This has been exacerbated by the workload increase caused by the COVID-19 pandemic. Major collapses in healthcare systems across the world during the peak of the pandemic led to calls for strategies to alleviate the increasing job attrition problem within the healthcare sector. This turnover may worsen given the overwhelming pressures experienced by the health workforce during the pandemic, and proactive measures should be taken to retain healthcare workers. This review aims to examine the factors affecting turnover intention among healthcare workers during the COVID-19 pandemic.

**Methods:**

A mixed studies systematic review was conducted. The PubMed, Embase, Scopus, CINAHL, Web of Science and PsycINFO databases were searched from January 2020 to March 2022. The Joanna Briggs Institute’s Critical Appraisal Tools and the Mixed Methods Appraisal Tool version 2018 were applied by two independent researchers to critically appraise the methodological quality. Findings were synthesised using a convergent integrated approach and categorised thematically.

**Results:**

Forty-three studies, including 39 quantitative, two qualitative and two mixed methods studies were included in this review. Eighteen were conducted in the Middle East, ten in the Americas, nine in the Asia–Pacific region and six in Europe. Nurses (*n* = 35) were included in the majority of the studies, while physicians (*n* = 13), allied health workers (*n* = 11) and healthcare administrative or management staff (*n* = 7) were included in a smaller proportion. Five themes emerged from the data synthesis: (1) fear of COVID-19 exposure, (2) psychological responses to stress, (3) socio-demographic characteristics, (4) adverse working conditions, and (5) organisational support.

**Conclusions:**

A wide range of factors influence healthcare workers’ turnover intention in times of pandemic. Future research should be more focused on specific factors, such as working conditions or burnout, and specific vulnerable groups, including migrant healthcare workers and healthcare profession minorities, to aid policymakers in adopting strategies to support and incentivise them to retain them in their healthcare jobs.

**Supplementary Information:**

The online version contains supplementary material available at 10.1186/s12960-022-00764-7.

## Background

The coronavirus disease 2019 (COVID-19) pandemic began in late 2019, with an estimated 400 000 000 infections and 6 200 000 deaths caused by the disease as of April 2022 [1]. Several coronavirus variants surfaced throughout the pandemic, resulting in repeated waves of widespread infections in countries worldwide. The severity of COVID-19 infections, coupled with the large number of cases, placed immense pressure on healthcare systems as high volumes of patients in need of acute treatment required hospitalisation; some regions suffered from high COVID-19 mortality rates due to shortages of medical workers and equipment [2].

A major contributing factor to the shortages seen in the health workforce is employee turnover [3]. In the context of the healthcare sector, turnover intention refers to the willingness of healthcare workers (HCWs) to leave their positions of employment for other positions in either the same or different professions [4]. The COVID-19 pandemic has caused an unprecedented wave of resignations. In the United States, the healthcare sector suffered a net loss of 460 000 workers between February 2020 and November 2021 [5]. A survey of 1000 American HCWs revealed that 18% of them left their jobs over the course of the pandemic, citing it as one of the driving factors behind their resignation [6]. Resignation rates of HCWs in Singapore spiked in 2021, and this was driven by both foreign workers looking to migrate, as well as local workers experiencing severe levels of burnout [7].

High turnover rates cause difficulty in staffing healthcare facilities adequately, which has several implications on the quality of care delivered to patients. Low nurse staffing is associated with increased patient mortality rates, as low nurse-to-patient ratios result in fewer nursing care hours available for each patient[8]. Other outcomes such as patient safety and quality of care are also adversely affected by healthcare understaffing, as higher quantities of care are left undone at the end of shifts[9]. Healthcare staff may suffer from stress and burnout when being overworked to compensate for low manpower which compromises their ability to deliver care, resulting in a higher risk of medical errors [10].

While some factors are known, turnover is caused by multiple factors, and a fuller understanding of these factors must be pursued if employers seek to reduce turnover. A search for systematic reviews published in the last 10 years examining turnover intention among HCWs on PubMed, PROSPERO and Google Scholar produced nine relevant systematic reviews [11–19]; none of which included studies that took place during the COVID-19 pandemic. One integrative review examined the COVID-19 pandemic’s impact on predictors of nurses’ turnover intention, but it included mainly pre-COVID-19 studies, which diminished the focus of the results regarding the current situation [20]. As evidenced by the increased turnover among HCWs, the COVID-19 pandemic has likely exacerbated many previously existing factors that affected turnover intention. The challenges facing human resource management in healthcare before and during the pandemic might differ. Considering the pressing healthcare turnover issue and the lack of reviews addressing turnover intention among HCWs during the COVID-19 pandemic, this mixed studies review aimed to examine factors affecting turnover intention in the context of the highly turbulent pandemic-focused healthcare environment.

## Methods

This review was conducted in accordance with the Joanna Briggs Institute (JBI) methodology for mixed methods systematic reviews using a convergent integrated approach [21].

### Search strategy

An initial search of PubMed was performed with free-text words addressing the review aims to identify relevant articles. Titles, abstracts, and keywords of these articles were analysed, which informed the development of an extensive search strategy, details of which are provided in Additional file 1. Key search terms included ‘healthcare worker’, ‘turnover’ and ‘COVID-19’. Reference lists of all studies selected for critical appraisal were also screened for additional studies. The PubMed, Embase, Scopus, CINAHL, Web of Science and PsycINFO databases were searched for studies published from January 2020 up to March 2022.

### Study selection

This review included studies that contained HCWs, following the definition by the World Health Organization as an occupation group consisting of doctors, nurses, and other professionals or supporting personnel, such as pharmacists, physiotherapists, and occupational therapists, that provide health services [22]. Studies that contained non-healthcare workers were also included if HCWs made up the majority of study participants. The review also included studies examining factors that affect turnover intention, as defined in the background [4], as an outcome. Quantitative, qualitative, and mixed methods studies of any design in the English language were included. Only peer-reviewed articles were considered to ensure high quality of included studies.

Following the search, all identified citations were collated and uploaded into EndNote 20 (Clavariate Analytics). Two reviewers (RP & PL) first independently screened the titles and abstracts for assessment against the inclusion criteria, followed by full-text articles. Full-text studies that did not meet the inclusion criteria were excluded. Any disagreements that arose between the two reviewers were resolved through discussion, with the assistance of a third reviewer (SL) where necessary.

### Assessment of methodological quality

The JBI checklist for analytical cross-sectional studies and the JBI checklist for qualitative research were used to appraise the included quantitative and qualitative studies, respectively [23]. The Mixed Methods Appraisal Tool (MMAT) version 2018 [24] was used to appraise mixed methods studies. Critical appraisal was performed by two independent reviewers, and disagreements were resolved through discussion. For this review, a low methodological quality refers to a score assigned to a study of less than 50%, a medium quality refers to one between 50 and 75%, and a high quality refers to one greater than 75%.

### Data extraction and synthesis

Full-text articles of eligible studies were retrieved and reviewed. To obtain relevant information that assisted in answering the review question, a customised data extraction template that included the origin and year of publication, study methodology and objective, occupations of participants and primary findings was used. Data was extracted independently by two reviewers (RP & PL), and any discrepancies observed were resolved through discussion with the assistance of a third reviewer (SL). Adhering to the JBI approach to mixed methods systematic reviews, a convergent integrated approach was adopted, where both quantitative and qualitative data were combined and synthesised simultaneously [21]. Quantitative data was first coded and presented in a textual descriptive form to allow for integration with qualitative data. A three-step thematic synthesis was then conducted [25]. Initial inductive codes were generated using line-by-line coding. These codes were organised into categories, forming descriptive themes. The reviewers then compared these descriptive themes with textual data from the studies, allowing analytical themes to emerge which were finalised through discussion among the two reviewers (RP & PL). All synthesised findings were presented in a narrative summary and categorised thematically.

## Results

### Search outcomes

**Fig. 1:**
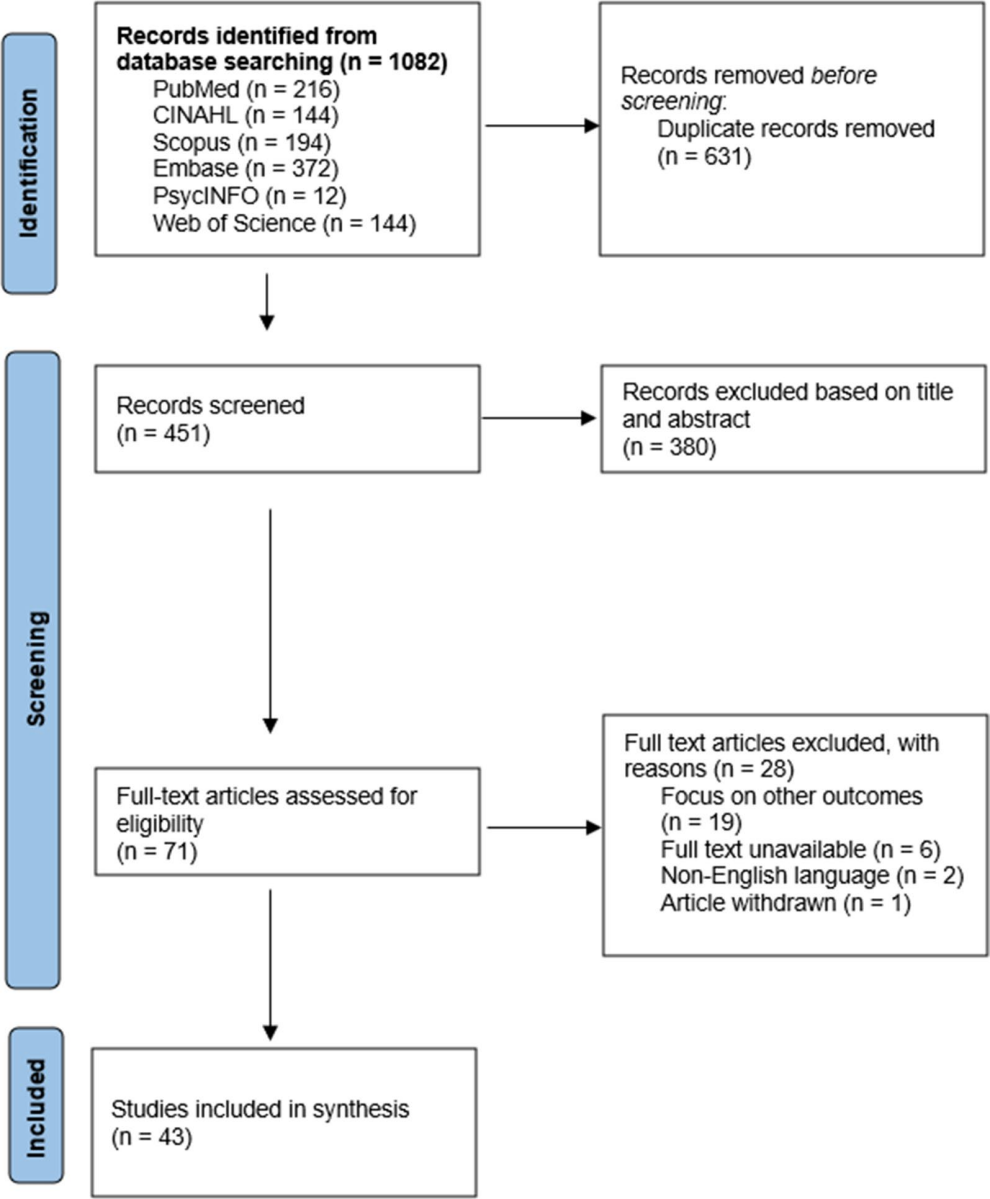
Modified PRISMA flow diagram

A total of 1,082 articles were retrieved. After removing 631 duplicates, 451 records were screened, based on titles and abstracts. Irrelevant records were removed, and 71 full-text articles were screened based on eligibility. A total of 43 articles [26–68] met the inclusion criteria and were included for the synthesis. The flow of the selection process is illustrated in Fig. 1, the modified Preferred Reporting Items for Systematic Reviews and Meta-Analyses (PRISMA) format.

### Study characteristics

Among the 43 included studies, there were 39 quantitative studies, two qualitative studies and two mixed methods studies. Eighteen studies were conducted in the Middle East, ten in the Americas, nine in the Asia–Pacific region and six in Europe. Nurses (*n* = 35) were included in a vast majority of the studies, while physicians (*n* = 13), allied health workers (*n* = 11) and healthcare administrative or management staff (*n* = 7) were included in a smaller proportion. Quantitative studies measured turnover intention with the Turnover Intention Scale (TIS-6) [69] or modified versions, Likert scales, or yes–no questions. Qualitative studies conducted semi-structured interviews with individual participants. Mixed methods studies used online questionnaires with both closed questions—Likert scales and yes–no questions—and open-ended questions. The sample sizes for quantitative and mixed methods studies ranged from 72 to 5,088 participants, while the sample sizes for qualitative studies were comparatively smaller and ranged from 10 to 19 participants. Refer to Table 1 for the study characteristics and summaries of their findings. The overall critical appraisal quality ratings of all included studies ranged from 71 to 100%, indicating medium to high methodological quality. Most studies that were unable to achieve high methodological quality did not use valid and reliable tools to measure turnover intention. Refer to Additional file 2 for the results of the quality appraisal.

**Table 1:** Study characteristics and findings from included studies

Author, country	Aim	Study design	Participants (*n*)	Data collection and instruments	Results	Themes derived
Abd-Ellatif et al. (2021), Egypt	To determine the effect of fear relating to COVID-19 on job satisfaction and turnover intention	Cross-sectional	Physicians (411)	Fear of COVID-19 Scale (FCV-19S), Job satisfaction scale/satisfaction with work scale (SWWS), Turnover intention scale (TIS-6)	Fear of COVID-19 is positively correlated with turnover intention Job satisfaction is negatively correlated with turnover intention	Fear of COVID-19 may contribute to turnover intention Job satisfaction may mitigate turnover intention
Alameddine et al. (2021a), Lebanon	To investigate the resilience levels, job satisfaction, and turnover intention of nurses	Cross-sectional	Nurses (265)	Connor-Davidson Resilience Scale (CD-RISC)	Resilience is negatively correlated with turnover intention	Resilience may mitigate turnover intention
Alameddine et al. (2021b), Lebanon	To determine the level of resilience in the nursing workforce and its relationship to burnout, intention to quit, and perceived COVID-19 risk	Cross-sectional	Nurses (511)	Connor-Davidson Resilience Scale (CD-RISC), Copenhagen Burnout Inventory (CBI)	Resilience is negatively correlated with turnover intention	Resilience may mitigate turnover intention
Alameddine et al. (2022), Lebanon	To determine the level of resilience and its relationship to burnout, job satisfaction, intention to quit, and changes in practice	Cross-sectional	Pharmacists (459)	Connor-Davidson Resilience Scale (CD-RISC)	Resilience is negatively correlated with turnover intention	Resilience may mitigate turnover intention
Alenazy et al. (2021), Australia/Saudi Arabia	To examine the relationship between perception of nursing practice environment (NPE), job satisfaction and intention to leave	Cross-sectional	Critical care nurses (152)	Practice Environment Scale of the Nursing Work Index (PES-NWI), Nursing Workplace Satisfaction Questionnaire (NWSQ), Turnover Intention Scale (TIS-6)	A positive NPE is negatively correlated with turnover intention Job satisfaction was not significantly related to turnover intention	Positive working conditions may mitigate turnover intention
Al-Mansour (2021), Saudi Arabia	To investigate the association between stress, social support and turnover intention among healthcare workers	Cross-sectional	Physicians (84) Nurses (134) Paramedical workers (84) Administrative staff (209)	Perceived Stress Scale-10 (PSS-10), Multidimensional Scale of Perceived Social Support (MSPSS)	Stress is positively correlated with turnover intention, with social support acting as a mediating factor	Stress and anxiety may contribute to turnover intention Social support may mitigate turnover intention
Blake et al. (2020), United Kingdom	To determine the effectiveness of well-being centres on employee well-being	Cross-sectional	Hospital employees (819)	Warwick Edinburgh Mental Well-being Scale (WEMWBS), Utrecht Work Engagement Scale (UWES-9)	There is no significant association between turnover intention and well-being centre access	The relationship between organisational support and turnover intention is inconclusive
Cimarolli et al. (2021), United States of America	To investigate the mediating role of employer communication and staff preparedness on turnover intention	Cross-sectional	Nursing home staff (1,683)	Questionnaires	Higher levels of COVID-19-related challenges are positively correlated with turnover intention Quality employer communication is indirectly and negatively associated with turnover intention, with job preparedness acting as a mediating factor	Fear of COVID-19 may contribute to turnover intention Organisational support may mitigate turnover intention
Cole et al. (2021), United States of America	To investigate if anxiety and stress from COVID-19 working conditions contribute to turnover intention	Cross-sectional	Nurses (111)	Questionnaires	Married and senior nurses are more likely to experience turnover intention Psychological stress and anxiety are positively correlated with turnover intention	Personal demographics may affect turnover intention Stress and anxiety may contribute to turnover intention
Cornish et al. (2021), Australia	To explore the intentions of emergency nurses to remain in or leave emergency nursing after the first year of the COVID-19 pandemic	Cross-sectional	Emergency nurses (398)	Questionnaires	Nurses who received COVID positive patients were more likely to experience turnover intention Nurses who did not feel connected to their colleagues, team or organisation were more likely to experience turnover intention	Fear of COVID-19 may contribute to turnover intention A lack of organisational support and social support may contribute to turnover intention
De los Santos and Labrague (2021), Philippines	To assess the impact of fear of COVID-19 on job stress and turnover intention among community nurses	Cross-sectional	Community nurses (385)	Fear of COVID-19 Scale (FCV-19S), Job Satisfaction Index (JSI), Job Stress Scale (JSS)	Fear of COVID-19 is positively correlated with turnover intention	Fear of COVID-19 may contribute to turnover intention
Elhanafy and El Hessewi (2021), Egypt	Effect of fear of COVID-19 pandemic on work satisfaction and turnover intentions of nurses	Cross-sectional	Nurses (210)	Fear of COVID-19 scale (FCV-19S), Job Satisfaction Index (JSI)	Fear of COVID-19 is positively correlated with turnover intention	Fear of COVID-19 may contribute to turnover intention
Fisher et al. (2021), United States of America	To explore factors that influenced the personal and professional well-being of care providers working in long-term care facilities	Qualitative descriptive	Certified nursing assistants (8) Nurses (4) Other (2)	Semi-structured interview	Workplace stressors such as high workloads and low staff morale are associated with turnover intention	Difficult working conditions may contribute to turnover intention
Fronda and Labrague (2022), Oman/Philippines	To examine the relationship between coronaphobia and frontline nurses’ organisational and professional turnover intention, and to assess whether social support and coping skills can buffer this relationship	Cross-sectional	Nurses (687)	Coronavirus Anxiety Scale (CAS), Brief Coping Skills Scale (BCS), Perceived Social Support Questionnaire (PSSQ)	Coronaphobia is positively related to turnover intention, with social support and coping skills acting as mediating factors	Fear of COVID-19 may contribute to turnover intention Social support may mitigate turnover intention
Hou et al. (2021), China	To assess the prevalence of turnover intention and explored associated factors on turnover intention among healthcare workers during the COVID-19 pandemic in China	Cross-sectional	Physicians (845) Nurses (344) Others (214)	Depression Anxiety Stress Scale − 21 (DASS-21), Perceived Social Support (PSS) Scale	Workers in secondary hospitals were more likely to experience turnover intention Workers with daily working hours of more than 12 h were more likely to experience turnover intention Workers who refused to volunteer for frontline roles were more likely to experience turnover intention Workers who experienced no change in patient relations were more likely to experience turnover intention Workers who experienced depression, low social support or a combination of psychosocial issues were more likely to experience turnover intention	Difficult working conditions may contribute to turnover intention Poor mental health may contribute to turnover intention
Khattak et al. (2021), Pakistan	To explore the moderating effect of leadership support in the relationship between fear of COVID-19, turnover intention and psychological distress in nurses	Cross-sectional	Nurses (380)	Fear of COVID-19 Scale (FCV-19S)	Fear of COVID-19 is positively correlated with turnover intention, with leadership support acting as a mediating factor	Fear of COVID-19 may contribute to turnover intention Organisational support may mitigate turnover intention
Labrague and de Los Santos (2021a), Oman/Philippines	To examine whether frontline nurses' fear of COVID-19 contributes to psychological distress, work satisfaction and intent to leave their organisation and the profession	Cross-sectional	Nurses (261)	Fear of COVID-19 Scale (FCV-19S), Job Satisfaction Index (JSI), Job Stress Scale (JSS)	Fear of COVID-19 is positively correlated with turnover intention	Fear of COVID-19 may contribute to turnover intention
Labrague and de Los Santos (2021b), Oman/Philippines	To examine the mediating role of resilience in the relationship between compassion fatigue and frontline nurses' job outcomes	Cross-sectional	Nurses (270)	Compassion Fatigue Scale (CFS), Brief Resilient Coping Skills (BRCS) scale	Compassion fatigue is positively correlated with turnover intention, with resilience acting as a mediating factor	Compassion fatigue may contribute to turnover intention Resilience may mitigate turnover intention
Labrague et al. (2021), Oman/Philippines	To assess the influence of COVID-19-associated discrimination on frontline nurses’ mental health and their intention to quit the nursing profession with resilience acting as a mediator	Cross-sectional	Nurses (259)	COVID-19-Associated Discrimination (CAD) scale, Brief Resilient Coping Skills (BRCS) scale, Mental Health Inventory (MHI)	COVID-19-associated discrimination is positively correlated with turnover intention, with resilience acting as a mediating factor	COVID-19-associated discrimination may contribute to turnover intention Resilience may mitigate turnover intention
Lavoie-Tremblay et al. (2021), Canada	To examine the influence of caring for COVID-19 patients on nurse’s perceptions of chronic fatigue, quality of care, satisfaction at work and intention to leave their organisation and the profession	Cross-sectional	Nurses (1705)	Transformational Leadership scale, Occupational Fatigue Exhaustion Recovery Scale (OFER)	High job demands and poor job resources are positively correlated with turnover intention Nurses who were infected or had team members who were infected with COVID-19 were more likely to experience turnover intention	Difficult working conditions may contribute to turnover intention Fear of COVID-19 may contribute to turnover intention
Lotfi et al. (2021), Iran	To determine turnover intention among operating room nurses during the COVID-19 outbreak and its association with perceived safety climate	Cross-sectional	Operating room nurses (190)	Anticipated Turnover Scale (ATS), Safety Climate Scale (SCS)	Perceived safety climate is negatively correlated with turnover intention	A strong safety climate may mitigate turnover intention
Magnavita et al. (2021a), Italy	To measure the perception of organisational justice and occupational stress and how these varied in relation to external factors, such as turnover intention	Cross-sectional	Intensive care physicians (120)	Colquitt questionnaire, Siegrist effort/reward imbalance model questionnaire	Physicians who put in high effort and perceived low rewards from work were more likely to experience turnover intention	Difficult working conditions may contribute to turnover intention A lack of organisational support may contribute to turnover intention
Magnavita et al. (2021b), Italy	To assess the well-being and mental health of the workers after the first 10 months of the pandemic and to evaluate the extent to which their attitude toward the pandemic had changed	Cross-sectional	Physicians (105) Nurses (47)	Goldberg Anxiety and Depression Scale (GADS), Sleep Condition Indicator (SCI-02)	Healthcare workers who put in high effort were more likely to experience turnover intention, with perceived rewards from work acting as a mediating factor	Difficult working conditions may contribute to turnover intention A lack of organisational support may contribute to turnover intention
Majeed et al. (2021), Pakistan	To investigate the mediating role of perceived fear of COVID-19 between perceived risk of COVID-19 and turnover intention	Cross-sectional	Nurses (103) Paramedical staff (59) Doctors (16)	Modified Perceived Vulnerability to Disease (PVD) questionnaire, Fear of COVID-19 Scale (FCV-19S)	Perceived risk of COVID-19 is positively correlated with turnover intention, with fear of COVID-19 acting as a mediating factor	Fear of COVID-19 may contribute to turnover intention
Mercado et al. (2022), United States of America	To examine the association between personal, work-related and contextual factors, and stress, burnout and turnover intention among healthcare workers in the COVID-19 pandemic	Cross-sectional	Medical staff (328) Allied health staff (379) Administrative staff (224) Others (36)	Mindful Self-Care Scale, Mental Health Continuum Short Form (MHC-SF), Healthcare Worker Exposure Response & Outcomes (HERO) Daily Impact Index, Pandemic Experiences and Perceptions Survey, Perceived Stress Scale (PSS-4)	Age and quality of work–life were negatively correlated with turnover intention	Personal demographics may affect turnover intention Working conditions may affect turnover intention
Mirzaei et al. (2021), Iran	To evaluate the relationship between turnover intention and job stressors in nurses during the COVID-19 outbreak	Cross-sectional	Nurses (479)	Impact of Event Scale-Revised (IES-R), General Health Questionnaire (GHQ), Turnover Intention Questionnaire, Job Content Questionnaire (JCQ)	Job stressors were positively correlated with turnover intention. Married nurses and male nurses were more likely to experience turnover intention Social support was negatively correlated with turnover intention	Difficult working conditions may contribute to turnover intention Personal demographics may affect turnover intention Social support may mitigate turnover intention
Naja et al. (2021), United Arab Emirates	To examine the conditions and changes in the work environment as well as resilience and its correlates among dieticians during the COVID-19 pandemic	Cross-sectional	Dieticians (371)	Connor–Davidson Resilience Scale (CD-RISC)	Resilience is negatively correlated with turnover intention	Resilience may mitigate turnover intention
Nashwan et al. (2021), Qatar	To compare nurses’ turnover intentions before and during COVID-19	Cross-sectional	Nurses (512)	Turnover Intention Scale (TIS-6)	Nurses were more likely to experience turnover intention during the COVID-19 pandemic as compared to beforehand	Fear of COVID-19 may contribute to turnover intention
Öğütlü et al. (2021), Turkey	To determine the relationship between stress, workload, and support in psychiatrists during the COVID-19 pandemic	Cross-sectional	Psychiatrists (217)	Copenhagen Burnout Inventory (CBI)	Burnout is positively correlated with turnover intention	Burnout may contribute to turnover intention
Özkan Şat et al. (2021), Turkey	To determine the relationship between nurses' exposure to violence and their professional commitment during the COVID-19 pandemic	Cross-sectional	Nurses (263)	Nursing Professional Commitment Scale (NPCS)	Nurses who experienced workplace violence were more likely to experience turnover intention Nurses who faced an increase in working hours, increase in workload and deployment to other departments were more likely to experience turnover intention	Difficult working conditions may contribute to turnover intention
Pförtner et al. (2021), Germany	To examine the relationship between long-term care managers’ intentions to quit their profession and demands that affect long-term care facilities during the COVID-19 pandemic	Cross-sectional	Long-term care facility managers (833)	Questionnaires	Increased pandemic-specific and general job demands are positively correlated with turnover intention	Difficult working conditions may contribute to turnover intention
Raso et al. (2021), United States of America	To describe the relationships between perceptions of the pandemic impact on nurses' intent to leave their current position and the profession	Cross-sectional	Nurses (5,088)	Questionnaires	Nurses who perceived greater pandemic impact on practice were more likely to experience turnover intention	Difficult working conditions may contribute to turnover intention
Riggan et al. (2021), United States of America	To assess the impact of the COVID-19 pandemic on obstetricians and gynaecologists	Mixed-methods	Obstetricians and gynaecologists (72)	Questionnaires (Likert scales & open-ended questions)	Burnout is associated with turnover intention	Burnout may contribute to turnover intention
Said and El-Shafei (2021), Egypt	To assess occupational stress, job satisfaction, and intent to leave among nurses dealing with suspected COVID-19 patients	Cross-sectional	Nurses (420)	Expanded Nursing Stress Scale (ENSS), Specific COVID-19-Associated Stressors (SCAS), McCloskey/Mueller Satisfaction Scale (MMSS)	Nurses with longer working hours and more night duties per week were more likely to experience turnover intention	Difficult working conditions may contribute to turnover intention
Schug et al. (2022), Germany	To examine the correlation between frequency and associated factors of sick leave and intention to quit among nurses	Cross-sectional	Nurses (757)	Effort-reward imbalance scale (ERI), PHQ-2 (Patient Health Questionnaire), Generalized Anxiety Disorder 2-item (GAD-2)	Perceived reward levels were negatively correlated with turnover intention Changing work departments during the pandemic was associated with increased turnover intention Depression levels were positively correlated with turnover intention	A lack of organisational support may contribute to turnover intention Difficult working conditions may contribute to turnover intention High levels of depression may contribute to turnover intention
Shah et al. (2022), Pakistan	To identify the association between job stress state anger, emotional exhaustion and job turnover intention	Cross-sectional	Nurses (318)	Maslach Burnout Inventory-General Survey (MBI)	COVID-19-related job stress, state anger and emotional exhaustion are positively correlated with turnover intention	Burnout may contribute to turnover intention
Sheppard et al. (2021), United States of America	To explore the level of moral distress among nurses	Cross-sectional	Nurses (107)	Measure of Moral Distress for Health Care Professionals (MMD-HP)	Nurses who perceived greater issues with patient safety and quality and work environment were more likely to experience turnover intention	Difficult working conditions may contribute to turnover intention
Sklar et al. (2021), United States of America	To examine the effects of work changes on burnout and subsequent turnover intentions in mental health providers, and how job and personal resources may have buffered the extent to which work changes due to COVID-19 impacted burnout	Cross-sectional	Outpatient mental health providers (93)	Copenhagen Work Burnout Inventory, Modified Michigan Organizational Assessment Questionnaire	Work changes were indirectly positively correlated with turnover intention through burnout, with organisational trust and perceived organisational support acting as mediating factors	Difficult working conditions may contribute to turnover intention Burnout may contribute to turnover intention Organisational support may mitigate turnover intention
Varasteh et al. (2021), Iran	To explore the factors affecting nurses’ intentions to leave or stay in their profession during the COVID-19 pandemic	Qualitative descriptive	Nurses (19)	Semi-structured interview	Professional commitment was associated with reduced turnover intention Fear of COVID-19 was associated with turnover intention A positive organisational atmosphere and organisational motivation was associated with reduced turnover intention	Professional commitment may mitigate turnover intention Fear of COVID-19 may contribute to turnover intention Organisational support may mitigate turnover intention
Wibowo and Paramita (2022), Indonesia	To investigate the impact of mindful and empathetic leadership on resilience and turnover intention, with self-regulation as a mediating variable	Cross-sectional	Nurses (188)	Modified Mindfulness Attention Awareness Scale (MAAS), Motivating Language Scale, Connor-Davidson Resilience Scale (CD-RISC)	Mindful leadership was associated with reduced turnover intention, with self-regulation acting as a mediating factor	Organisational support may mitigate turnover intention Self-regulation may mitigate turnover intention
Wood et al. (2021), United Kingdom	To understand the experiences of advanced practice nurses during the COVID-19 pandemic in relation to safety, shortages and retention	Mixed-methods	Nurses (124)	Questionnaires (Likert scales & open-ended questions)	PPE shortages and fear of COVID-19 were both associated with turnover intention	Fear of COVID-19 may contribute to turnover intention
Yáñez et al. (2020), Peru	To assess the anxiety, distress, and turnover intention of healthcare workers during the COVID-19 pandemic	Cross-sectional	Hospital technicians (80) Nurses (63) Pharmacists (63) Physicians (53) Hospital volunteers (20) Others (24)	Generalized Anxiety Disorder (GAD-7) scale, Kessler Psychological Distress scale (K6)	Younger workers were more likely to experience turnover intention	Personal demographics may affect turnover intention
Yang et al. (2021), China	To elucidate the effects of workplace violence on turnover intention among Chinese health care workers, and to identify the potential mediators in this relationship	Cross-sectional	Doctors, nurses and allied health workers (1,063)	Perceived Social Support (PSS) scale, Depression Anxiety Stress Scales-21 (DASS-21)	Workplace violence was positively correlated with turnover intention, with social support and mental health acting as mediating factors	Difficult working conditions may contribute to turnover intention Social support may mitigate turnover intention General stress and anxiety may contribute to turnover intention

Data was categorised into five themes that emerged during the extraction process: (1) fear of COVID-19 exposure, (2) psychological responses to stress, (3) socio-demographic characteristics, (4) adverse working conditions, and (5) organisational support.

### Data synthesis

#### Fear of COVID-19 exposure

The most prevalent theme was fear of COVID-19 exposure, which had the greatest number of studies reporting about it. A total of 12 studies revealed a positive correlation or association between fear of COVID-19 and turnover intention. Six quantitative studies used the Fear of COVID-19 Scale (FCV-19S) as an instrument to measure the psychological impact of exposure to COVID-19 on HCWs [26, 36, 37, 41, 42, 49], while one study used the Coronavirus Anxiety Scale (CAS) [39], with all studies finding a positive correlation between fear of COVID-19 exposure and turnover intention. Two studies from Iran and the United Kingdom also found fear of COVID-19 exposure to be associated with turnover intention through qualitative results [64, 66]. Nurses were at a greater risk of experiencing turnover intention during the COVID-19 pandemic compared to pre-pandemic times [53]. Nurses who were infected or had team members who were infected with COVID-19 [45] or received COVID-19 positive patients [35] were more likely to experience turnover intention. Within nursing home staff, facing increased COVID-19-related challenges, such as a lack of personal protective equipment (PPE) and increased risk of COVID-19 transmission, were indirectly and positively associated with turnover [33].

#### Psychological responses to stress

A variety of psychological responses to stress displayed by HCWs experiencing turnover intention was observed in several studies Two studies from Saudi Arabia and the United States discovered that high levels of psychological stress and anxiety were linked to HCWs exhibiting higher degrees of turnover intention [31, 34], while a study from Germany linked higher levels of depression to greater turnover intention [60]. Burnout was another key element that was associated with increased turnover intention [54, 58, 61, 63]. Furthermore, two Chinese studies found that HCWs were at a greater chance of experiencing turnover intention if they were suffering from poor mental health or a combination of psychosocial issues [40, 68]. A study that surveyed nurses from the Philippines found that turnover intention was positively correlated with compassion fatigue [43]. In addition, COVID-19-associated discrimination was positively correlated with turnover intention, as it contributed to aggravating factors, such as poor mental health and burnout [44]. On the other hand, resilience within HCWs was found to be negatively correlated with turnover intention [27–29, 52], with two studies concluding that it acted as a protective mechanism against factors that contributed to turnover intention [43, 44]. Professional commitment and job satisfaction were other psychological factors that were associated with reduced turnover intention within nurses and physicians, respectively [26, 64].

#### Socio-demographic characteristics

Three studies noted that certain socio-demographic characteristics significantly influenced the likelihood of HCWs experiencing turnover intention [34, 51, 67]. Two studies reported that married nurses were more likely to experience turnover intention [34, 51]. Mirzaei et al. [51] also noted that male nurses experienced turnover intention at a higher rate. While two American studies found that seniority in nurses was associated with greater turnover intention [34, 50], this was in contrast to findings in a study from Peru [67], which reported that younger HCWs were more prone to experiencing turnover intention. Social affiliation was also identified by some studies to impact turnover intention, as nurses with strong ties to friends and family perceived greater social support. Three studies determined that social support was able to act as a mediating factor in reducing turnover intention among nurses [31, 39, 68]. A direct correlation between social support and reduced turnover intention was also established by an Iranian study [51]. Conversely, an Australian study found that nurses who did not feel connected to their colleagues or team were at greater risk of experiencing turnover intention [35].

#### Adverse working conditions

The COVID-19 pandemic placed heavy pressure on healthcare systems, resulting in excessive job demands and tumultuous work environments. Increases in workload were widely found to be positively correlated with turnover intention among HCWs [38, 45, 55, 56]. In addition, HCWs who worked long hours, or faced an increase in working hours due to the pandemic, were more prone to experiencing turnover intention [40, 50, 55, 59]. Mirzaei et al. [51] found that job stressors were positively correlated with turnover intention. Two studies from Turkey and China found that being subjected to workplace violence was associated with increased turnover intention among HCWs, as traumatic experiences accelerated burnout and mental health deterioration [55, 68]. HCWs were also more likely to experience turnover intention if they faced changes at work [63] or deployment to other departments [55, 60]. A study from Iran found that the perceived safety climate was negatively correlated with turnover intention [46]. In addition, nurses who perceived greater issues with patient safety and quality, as well as work environment, were at a greater risk of experiencing turnover intention, as they were more substantially affected by moral distress [62]. Two Italian studies found that HCWs who put in higher levels of effort in their work also had a higher chance of experiencing turnover intention [47, 48]. Other factors that were associated with turnover intention among HCWs include poor job resources [45], low staff morale [45] and perceived high pandemic impact on practice [57]. Conversely, a study from Saudi Arabia found that a positive nursing practice environment was negatively correlated with turnover intention [30].

#### Organisational support

Several domains of organisational support were examined by studies and were found to impact turnover intention among HCWs in various ways. Organisational trust and perceived organisational support were core factors that protected nurses from increased turnover intention [63]. Other factors, such as quality employer communication and job preparedness, were also associated with decreased turnover intention among nursing home staff [33]. Moreover, leadership support was linked to decreased turnover intention among nurses [41, 65]. A qualitative study from Iran discovered that both a positive organisational atmosphere and organisational motivation were associated with reduced turnover intention among nurses [64]. In contrast, Australian nurses who did not feel connected to their organisation were more likely to experience turnover intention [35]. HCWs who perceived low rewards from work were also more prone to experiencing turnover intention [47, 48, 60]. A study from the United Kingdom did not find any significant association between turnover intention and access to well-being centres [32].

## Discussion

Findings of this study identified multiple factors influencing turnover intention among HCWs during the COVID-19 pandemic. The five emerged themes encompassed factors ranging from individual, interpersonal, job-related, and organisational determinants, and many of which were known factors prior to the pandemic [11–19]. However, the theme ‘fear of COVID-19 exposure’ was unique and specific to this pandemic.

HCWs’ fear of COVID-19 infection emerged as the most prevalent theme, especially during the initial stage of uncertainty and limited understanding of the virulence, transmission, and health management of COVID-19. Prior to the widespread deployment of vaccines, COVID-19 was potentially a life-threatening infection. Some HCWs were at high risk of exposure while tending infected patients in the face of shortages of fundamental resource, such as PPE [70]. The possibility of spreading COVID-19 to family members also created a concerning overlap between nurses’ professional and personal lives [71]. Several countries, such as Singapore and Hong Kong, which experienced the 2002–2004 severe acute respiratory syndrome (SARS) outbreak, reported higher pandemic preparedness and response resources, e.g., PPE and negative pressure rooms in intensive care units (ICU), and this could have better mitigated HCWs’ fears regarding COVID-19 [72]. Other strategies for addressing fear include clear, trustworthy, and timely COVID-19-related interpersonal, institutional and systemwide communication, to avoid disparities in understanding and reduce work-related stress among HCWs [73, 74].

The adverse working conditions as a result of pandemic emergency responses contribute to HCWs’ turnover intention. With the increase in patients requiring ICU care and mass testing services, HCWs faced increased workloads, higher nurse-to-patient ratios and deployment to areas requiring more staffing [75]. In particular, mass staff deployment means that members of a healthcare team are not familiar with each other, which can impact interprofessional collaboration [76]. This results in continuous tensions between healthcare professionals attempting to maintain patient safety under trying conditions [77]. In addition, the limitations posed by public health measures forced HCWs to quickly adapt new and frequently changing protocols and establish new workstreams, such as delivering care through telehealth [78]. Expectations of maintaining high standards of quality care remained, despite HCWs having to adapt to fluid and demanding working environments, facing new challenges and learning new skills [79]. Inevitably, HCWs experienced immense pressure and eventually burnout, which is a major contributor to turnover intention [55]. Burnout accompanies the progression of emotional overburdening, deteriorating mental well-being, and job dissatisfaction, which may drive HCWs to conclude that resignation is their best option [80]. In hindsight, these phenomena could be alleviated by judicious considerations of the consequences and the initiation of mitigating health policy measures.

Resilience within HCWs was observed to be a vital protective factor against turnover intention, as it enabled them to better respond to the disruptions that occurred during the pandemic. Staff who exhibit resilience are able to effectively use coping skills that reduce the psychological burden of treating COVID-19 patients [81]. However, when confronted with extended turmoil, HCWs found it increasingly difficult to remain unaffected while carrying out their everyday duties. High levels of psychological and moral distress were experienced by HCWs as they witnessed patient death and suffering on a massively increased scale during the pandemic [82]. To make matters worse, frontline HCWs around the world endured episodes of harassment and violence at the hands of members of the public who held irrational beliefs about the transmissibility of COVID-19 [83]. HCWs of Chinese and other Asian ethnicities were subjected to harsh COVID-19-related racial discrimination, adding to the difficulties experienced during the pandemic [68, 84]. Future studies could look into the impact of racism on turnover intention among HCWs. It is important to provide HCWs with opportunities to ‘let off steam’, obtain peer support, allow access to keep in contact with family and friends, and perform daily quick check-ins and check-outs to monitor their health status, support them emotionally and bolster their resilience [85]. At the organisational level, ensuring the provision of accessible and optimal professional psychosocial support, such as by having a multi-disciplinary psychosocial team, 24/7 hotline and efficient referral system could also be facilitated [85]. Measures to provide psychosocial support and mitigate secondary stressors related to the basic needs of life (e.g., childcare, grocery shopping) for HCWs in isolation or quarantine should also not be neglected [86].

Several studies in this review investigated different domains of organisational support. In essence, any form of organisational support that can be perceived by HCWs will empower them to adapt to the demands of their work and motivate them to perform their duties to the best of their ability [87]. During a health crisis leadership is challenging, but it is in times of crisis that the visibility and roles of leaders become apparent and provide opportunities for healthcare teams to grow and develop stronger relationships. Apart from communication and empowerment, Walton and colleagues [86] recommended the importance of understanding the humanity of the situation and exercising humility in demonstrating role responsibilities among healthcare leaders in supporting their teams through the pandemic. Similarly, being present was especially powerful in boosting nurses’ morale—high visibility of nurse leadership was evident during instances, where nurse leaders were physically present in COVID-19 treatment units to assist in various roles, building confidence and encouraging staff nurses to continue working [88].

Interestingly, this review did not identify financial renumeration as a factor contributing to turnover intention in times of the COVID-19 pandemic. Similarly, in another systematic review that identified barriers to manpower retention during health emergencies, poor leadership communication, emotional support and family worries were most commonly reported, while lacking budget in training, salaries and compensation of personnel were least reported [89]. While it is unclear what contributes to such a finding, financial renumeration is an important factor that impacts the livelihood of HCWs. Reimbursing HCWs to continue their professional education, establishing career ladders with attractive compensation progression, and maintaining salaries that are reasonably comparable with other local healthcare facilities are recommended forms of financial expenditure that can reduce turnover intention [90].

This review also identified that certain socio-demographic characteristics were associated with turnover intention. Married nurses struggled to achieve a work–life balance, especially those with children, as the pandemic caused increased childcare needs as a result of the implementation of virtual learning during lockdowns [91]. The inconsistent finding on impact of age on turnover intention was likely attributed by the different pandemic-related challenges faced by young and senior HCWs. Older HCWs were more susceptible to severe COVID-19 infection and faced age-related discrimination, while the less experienced younger HCWs had lesser personal resources and might be not as capable of protecting their well-being via self-regulation [92]. Special attention should thus be given to young/inexperienced and conversely, older HCWs. Social support protects against mental health stressors, acting similar to resilience in helping HCWs cope better during such difficult times [93]. Other vulnerable groups not identified in our review included the migrant HCWs who have been separated from their families since the start of pandemic and were unable to visit their country of origin due to travel or hospital administration restrictions [94].

Prior to the pandemic, many countries were facing healthcare workforce shortages, and the pandemic brought added challenges for healthcare stakeholders in retaining the current workforce. Nonetheless, the pandemic has also stirred sympathy and gratitude toward the plight of HCWs among citizens, providing a crucial opportunity for policymakers to justify and commit the resources required to achieve meaningful healthcare reform [95]. Ultimately, it is in the interest of public health stakeholders to capitalise on this opportunity to re-evaluate the support and compensation of HCWs, particularly in countries that face an increased demand for health services due to ageing populations.

### Implications for future research

While it would be valuable to healthcare leaders and policymakers for identifying the significant contributing factors impacting HCWs’ turnover intention in times of pandemic crisis, it was not the intent of this review to identify the strength of relationships between them, or the changes in weighting of these factors. Some of these existing factors might have become more important or less important during the COVID-19 pandemic. Future quantitative works could examine this. Future research conducted in times of crises could also focus on specific factors, such as working conditions or burnout, to elucidate the main drivers that influence them and how to better support or incentivise HCWs to stay in their jobs. In addition, specific vulnerable population groups, e.g., migrant HCWs and healthcare profession minorities, could be examined as they may face different and unique challenges in their personal lives and lines of work, respectively. As border controls inevitably ease over time, international travel will likely return to pre-pandemic levels and researchers could also investigate global labour market trends, such as migration, when analysing data related to HCWs turnover. While the fear of COVID-19 is a pandemic-specific factor, the remaining factors identified in this review were already present before the pandemic and were exacerbated by the extreme conditions of the pandemic; it is unclear whether these factors will diminish as the pandemic wanes, and thus future research can also serve to investigate the persistence of these factors.

### Strengths and limitations

This review captured studies conducted across a wide range of countries with different cultural and social contexts. However, it did not include grey literature and studies published in non-English languages. The methodological quality of the studies included in this review ranged from medium to high, but some studies did not account for confounding factors in their analyses, which would likely influence the reliability of their results. While the included studies focused on turnover intention instead of actual turnover, there is evidence that these are correlated [69]. Due to the lack of heterogeneity across the included studies, a meta-analysis could not be performed.

## Conclusions

In this review, we have provided an extensive overview of factors contributing to turnover intention among HCWs during the COVID-19 pandemic. Although it is unclear if some of the pandemic-specific factors identified will diminish over time as the pandemic ebbs, our findings highlighted the importance of acknowledging and addressing these factors to prevent further aggravation of the turnover issue. In the wake of the overwhelming pressures experienced by the health workforce in the past 2 years, this turnover may worsen, and proactive measures should be taken to retain HCWs. Future research should be more focused on specific factors, such as working conditions or burnout, and specific vulnerable groups, including migrant HCWs and healthcare profession minorities, to aid policymakers in adopting strategies to support them and incentivise them to retain them in their healthcare jobs.

## Supplementary Information

**Additional file 1. **Detailed search strategy for all databases.

**Additional file 2. **Quality appraisal of included studies.

## Data Availability

Data are available from the corresponding author upon request.

## Abbreviations

COVID-19: Coronavirus disease 2019
HCWs: Healthcare workers
ICU: Intensive care units
JBI: Joanna Briggs Institute

## References

[1] World Health Organization. WHO COVID-19 dashboard. 2022. https://covid19.who.int/.

[2] Armocida B, Formenti B, Ussai S, Palestra F, Missoni E (2020). The Italian health system and the COVID-19 challenge. Lancet Public Health.

[3] Dawson AJ, Stasa H, Roche MA, Homer CSE, Duffield C (2014). Nursing churn and turnover in Australian hospitals: nurses perceptions and suggestions for supportive strategies. BMC Nurs.

[4] Hayes LJ, O’Brien-Pallas L, Duffield C, Shamian J, Buchan J, Hughes F (2006). Nurse turnover: a literature review. Int J Nurs Stud.

[5] Bureau of Labor Statistics. The Employment Situation—October 2020. 2020 [cited 2022 Mar 3]. https://www.dol.gov/newsroom/economicdata/empsit_11062020.pdf.

[6] Galvin G. Nearly 1 in 5 Health care workers have quit their jobs during the pandemic. Morning Consult [Internet]. 2021 [cited 2022 Mar 3]. https://morningconsult.com/2021/10/04/health-care-workers-series-part-2-workforce/.

[7] Tan C. More healthcare workers in S’pore quit amid growing fatigue as Covid-19 drags on. The Straits Times[Internet]. 2021 [cited 2022 Mar 3]. https://www.straitstimes.com/singapore/politics/more-healthcare-workers-inspore-resigning-amid-growing-fatigue-as-covid-19-drags.

[8] Liang Y-W, Chen W-Y, Lee J-L, Huang L-C (2012). Nurse staffing, direct nursing care hours and patient mortality in Taiwan: the longitudinal analysis of hospital nurse staffing and patient outcome study. BMC Health Serv Res.

[9] Cho E, Lee N-J, Kim E-Y, Kim S, Lee K, Park K-O (2016). Nurse staffing level and overtime associated with patient safety, quality of care, and care left undone in hospitals: a cross-sectional study. Int J Nurs Stud.

[10] Montgomery A, Todorova I, Baban A, Panagopoulou E (2013). Improving quality and safety in the hospital: the link between organizational culture, burnout, and quality of care. Br J Health Psychol.

[11] Cowden T, Cummings G, Profetto-McGrath J (2011). Leadership practices and staff nurses' intent to stay: a systematic review. J Nurs Manag.

[12] Daouk-Öyry L, Anouze AL, Otaki F, Dumit NY, Osman I (2014). The JOINT model of nurse absenteeism and turnover: a systematic review. Int J Nurs Stud.

[13] Halter M, Boiko O, Pelone F, Beighton C, Harris R, Gale J (2017). The determinants and consequences of adult nursing staff turnover: a systematic review of systematic reviews. BMC Health Serv Res.

[14] Lai GC, Taylor EV, Haigh MM, Thompson SC (2018). Factors affecting the retention of indigenous australians in the health workforce: a systematic review. Int J Environ Res Public Health.

[15] Lartey S, Cummings G, Profetto-McGrath J (2014). Interventions that promote retention of experienced registered nurses in health care settings: a systematic review. J Nurs Manag.

[16] Lee J (2022). Nursing home nurses' turnover intention: a systematic review. Nurs Open.

[17] Russell D, Mathew S, Fitts M, Liddle Z, Murakami-Gold L, Campbell N (2021). Interventions for health workforce retention in rural and remote areas: a systematic review. Hum Resour Health.

[18] Shen X, Jiang H, Xu H, Ye J, Lv C, Lu Z (2020). The global prevalence of turnover intention among general practitioners: a systematic review and meta-analysis. BMC Fam Pract.

[19] Yildiz B, Yildiz H, Ayaz AO (2021). Relationship between work-family conflict and turnover intention in nurses: a meta-analytic review. J Adv Nurs.

[20] Falatah R (2021). The impact of the coronavirus disease (COVID-19) pandemic on nurses’ turnover intention: an integrative review. Nurs Rep..

[21] Lizarondo L, Stern C, Carrier J, Godfrey C, Rieger K, Salmond S, et al. Chapter 8: Mixed Methods Systematic Reviews. JBI Manual for Evidence Synthesis: JBI; 2020. 10.46658/JBIMES-20-09. 10.11124/JBISRIR-D-19-00169 32813460

[22] World Health Organization. Classification of health workforce statistics. 2010. https://www.who.int/hrh/statistics/Health_workers_classification.pdf.

[23] Joanna Briggs Institute. Critical Appraisal Tools. 2021. https://jbi.global/critical-appraisaltools.

[24] Hong QN, Fàbregues S, Bartlett G, Boardman F, Cargo M, Dagenais P (2018). The Mixed Methods Appraisal Tool (MMAT) version 2018 for information professionals and researchers. Educ Inf.

[25] Thomas J, Harden A (2008). Methods for the thematic synthesis of qualitative research in systematic reviews. BMC Med Res Methodol.

[26] Abd-Ellatif EE, Anwar MM, AlJifri AA, El Dalatony MM (2021). Fear of COVID-19 and its impact on job satisfaction and turnover intention among Egyptian physicians. Saf Health Work.

[27] Alameddine M, Bou-Karroum K, Ghalayini W, Abiad F (2021). Resilience of nurses at the epicenter of the COVID-19 pandemic in Lebanon. Int J Nurs Sci.

[28] Alameddine M, Clinton M, Bou-Karroum K, Richa N, Doumit MAA (2021). Factors associated with the resilience of nurses during the COVID-19 pandemic. Worldviews Evid Based Nurs.

[29] Alameddine M, Bou-Karroum K, Hijazi MA (2022). A national study on the resilience of community pharmacists in Lebanon: a cross-sectional survey. J Pharm Policy Pract..

[30] Alenazy FS, Dettrick Z, Keogh S (2021). The relationship between practice environment, job satisfaction and intention to leave in critical care nurses. Nurs Crit Care.

[31] Al-Mansour K (2021). Stress and turnover intention among healthcare workers in Saudi Arabia during the time of COVID-19: Can social support play a role?. PLoS ONE.

[32] Blake H, Yildirim M, Wood B, Knowles S, Mancini H, Coyne E (2020). Covid-well: evaluation of the implementation of supported wellbeing centres for hospital employees during the COVID-19 pandemic. Int J Environ Res Public Health.

[33] Cimarolli VR, Bryant NS, Falzarano F, Stone R (2022). Job resignation in nursing homes during the COVID-19 pandemic: the role of quality of employer communication. J Appl Gerontol.

[34] Cole A, Ali H, Ahmed A, Hamasha M, Jordan S (2021). Identifying patterns of turnover intention among alabama frontline nurses in hospital settings during the covid-19 pandemic. J Multidiscip Healthc.

[35] Cornish S, Klim S, Kelly AM (2021). Is COVID-19 the straw that broke the back of the emergency nursing workforce?. Emerg Med Australas.

[36] De los Santos JAA, Labrague LJ (2021). The impact of fear of COVID-19 on job stress, and turnover intentions of frontline nurses in the community: a cross-sectional study in the Philippines. Traumatology.

[37] Elhanafy EY, El Hessewi GS (2021). Effect of fear of COVID-19 pandemic on work satisfaction and turnover intentions of nurses. Egypt Nurs J.

[38] Fisher E, Cárdenas L, Kieffer E, Larson E (2021). Reflections from the "Forgotten Front Line": a qualitative study of factors affecting wellbeing among long-term care workers in New York City during the COVID-19 pandemic. Geriatr Nurs.

[39] Fronda DC, Labrague LJ (2022). Turnover intention and coronaphobia among frontline nurses during the second surge of COVID-19: The mediating role of social support and coping skills. J Nurs Manag.

[40] Hou H, Pei YF, Yang YM, Lu LL, Yan WJ, Gao XY (2021). Factors associated with turnover intention among healthcare workers during the coronavirus disease 2019 (COVID-19) pandemic in China. Risk Manag Healthc Policy.

[41] Khattak SR, Saeed I, Rehman SU, Fayaz M (2021). Impact of fear of COVID-19 pandemic on the mental health of nurses in Pakistan. J Loss Trauma.

[42] Labrague LJ, de Los Santos JAA (2021). Fear of COVID-19, psychological distress, work satisfaction and turnover intention among frontline nurses. J Nurs Manag.

[43] Labrague LJ, Santos JAA de los. Resilience as a mediator between compassion fatigue, nurses' work outcomes, and quality of care during the COVID-19 pandemic. Appl Nurs Res. 2021; 10.1016/j.apnr.2021.151476 10.1016/j.apnr.2021.151476 PMC8448586 34544570

[44] Labrague LJ, De Los Santos JAA, Fronda DC (2021). Perceived COVID-19-associated discrimination, mental health and professional-turnover intention among frontline clinical nurses: the mediating role of resilience. Int J Ment Health Nurs.

[45] Lavoie-Tremblay M, Gélinas C, Aubé T, Tchouaket E, Tremblay D, Gagnon MP (2022). Influence of caring for COVID-19 patients on nurse's turnover, work satisfaction and quality of care. J Nurs Manag.

[46] Lotfi M, Akhuleh OZ, Judi A, Khodayari M (2022). Turnover intention among operating room nurses during the COVID-19 outbreak and its association with perceived safety climate. Perioper Care Oper Room Manag.

[47] Magnavita N, Soave PM, Antonelli M (2021). A one-year prospective study of work-related mental health in the intensivists of a COVID-19 hub hospital. Int J Environ Res Public Health.

[48] Magnavita N, Soave PM, Antonelli M (2021). Prolonged stress causes depression in frontline workers facing the COVID-19 pandemic-a repeated cross-sectional study in a COVID-19 Hub-Hospital in Central Italy. Int J Environ Res Public Health.

[49] Majeed M, Irshad M, Bartels J (2021). The interactive effect of COVID-19 risk and hospital measures on turnover intentions of healthcare workers: A time-lagged study. Int J Environ Res Public Health.

[50] Mercado M, Wachter K, Schuster RC, Mathis CM, Johnson E, Davis OI (2022). A cross-sectional analysis of factors associated with stress, burnout and turnover intention among healthcare workers during the COVID-19 pandemic in the United States. Health Soc Care Community.

[51] Mirzaei A, Rezakhani Moghaddam H, Habibi SA (2021). Identifying the predictors of turnover intention based on psychosocial factors of nurses during the COVID-19 outbreak. Nurs Open.

[52] Naja F, Radwan H, Cheikh Ismail L, Hashim M, Rida WH, Abu Qiyas S (2021). Practices and resilience of dieticians during the COVID-19 pandemic: a national survey in the United Arab Emirates. Hum Resour Health.

[53] Nashwan AJ, Abujaber AA, Villar RC, Nazarene A, Al-Jabry MM, Fradelos EC (2021). Comparing the impact of covid-19 on nurses’ turnover intentions before and during the pandemic in qatar. J Pers Med..

[54] Ogutlu H, McNicholas F, Turkcapar H (2021). STRESS AND BURNOUT IN PSYCHIATRISTS IN TURKEY DURING COVID-19 PANDEMIC. Psychiatr Danub.

[55] Özkan Şat S, Akbaş P, Yaman SŞ (2021). Nurses' exposure to violence and their professional commitment during the COVID-19 pandemic. J Clin Nurs.

[56] Pförtner TK, Pfaff H, Hower KI (2021). Will the demands by the covid-19 pandemic increase the intent to quit the profession of long-term care managers? A repeated cross-sectional study in Germany. J Public Health (Oxf).

[57] Raso R, Fitzpatrick JJ, Masick K (2021). Nurses' intent to leave their position and the profession during the COVID-19 pandemic. J Nurs Adm.

[58] Riggan KA, Reckhow J, Allyse MA, Long M, Torbenson V, Rivera-Chiauzzi EY (2021). Impact of the COVID-19 Pandemic on Obstetricians/Gynecologists. MCP.

[59] Said RM, El-Shafei DA (2021). Occupational stress, job satisfaction, and intent to leave: nurses working on front lines during COVID-19 pandemic in Zagazig City. Egypt Environ Sci Pollut Res Int.

[60] Schug C, Geiser F, Hiebel N, Beschoner P, Jerg-Bretzke L, Albus C (2022). Sick leave and intention to quit the job among nursing staff in German hospitals during the COVID-19 pandemic. Int J Environ Res Public Health.

[61] Shah SHA, Haider A, Jindong J, Mumtaz A, Rafiq N (2022). The impact of job stress and state anger on turnover intention among nurses during COVID-19: the mediating role of emotional exhaustion. Front Psychol.

[62] Sheppard KN, Runk BG, Maduro RS, Fancher M, Mayo AN, Wilmoth DD (2022). Nursing moral distress and intent to leave employment during the COVID-19 pandemic. J Nurs Care Qual.

[63] Sklar M, Ehrhart MG, Aarons GA (2021). COVID-related work changes, burnout, and turnover intentions in mental health providers: a moderated mediation analysis. Psychiatr Rehabil J.

[64] Varasteh S, Esmaeili M, Mazaheri M (2021). Factors affecting Iranian nurses' intention to leave or stay in the profession during the COVID-19 pandemic. Int Nurs Rev.

[65] Wibowo A, Paramita W (2021). Resilience and turnover intention: the role of mindful leadership, empathetic leadership, and self-regulation. J Leadersh Organ Stud..

[66] Wood E, King R, Senek M, Robertson S, Taylor B, Tod A (2021). UK advanced practice nurses' experiences of the COVID-19 pandemic: a mixed-methods cross-sectional study. BMJ Open.

[67] Yáñez JA, Afshar Jahanshahi A, Alvarez-Risco A, Li J, Zhang SX (2020). Anxiety, distress, and turnover intention of healthcare workers in Peru by their distance to the epicenter during the COVID-19 crisis. Am J Trop Med Hyg.

[68] Yang Y, Wang P, Kelifa MO, Wang B, Liu M, Lu L (2021). J Nurs Manag.

[69] Bothma CFC, Roodt G (2013). The validation of the turnover intention scale. SA J Hum Resour Manag.

[70] Ehrlich H, McKenney M, Elkbuli A (2020). Protecting our healthcare workers during the COVID-19 pandemic. Am J Emerg Med.

[71] Tang CJ, Lin YP, Chan EY (2021). 'From Expert to Novice', perceptions of general ward nurses on deployment to outbreak intensive care units during the COVID-19 pandemic: a qualitative descriptive study. J Clin Nurs.

[72] Rajamani A, Subramaniam A, Shekar K, Haji J, Luo J, Bihari S (2021). Personal protective equipment preparedness in Asia-Pacific intensive care units during the coronavirus disease 2019 pandemic: a multinational survey. Aust Crit Care.

[73] Cawcutt KA, Starlin R, Rupp ME (2020). Fighting fear in healthcare workers during the COVID-19 pandemic. Infect Control Hosp Epidemiol.

[74] Teo I, Chay J, Cheung YB, Sung SC, Tewani KG, Yeo LF (2021). Healthcare worker stress, anxiety and burnout during the COVID-19 pandemic in Singapore: a 6-month multi-centre prospective study. PLoS ONE.

[75] Hoogendoorn ME, Brinkman S, Bosman RJ, Haringman J, de Keizer NF, Spijkstra JJ (2021). The impact of COVID-19 on nursing workload and planning of nursing staff on the Intensive Care: a prospective descriptive multicenter study. Int J Nurs Stud.

[76] Lin YP, Chan LYC, Chan EY (2022). Tenacious team, precarious patient: a phenomenological inquiry into interprofessional collaboration during ICU resuscitations. J Adv Nurs.

[77] Lin YP, Tang CJ, Tamin VA, Tan LYC, Chan EY (2021). The hand-brain-heart connection: ICU nurses' experience of managing patient safety during COVID-19. Nurs Crit Care.

[78] Young M. Pandemic stress, burnout contribute to nursing pipeline shortage. Relias Media. 2021. https://www.reliasmedia.com/articles/148193-pandemic-stress-burnout-contribute-to-nursingpipeline-shortage.

[79] Akgün KM, Collett D, Feder SL, Shamas T, Schulman-Green D (2020). Sustaining frontline ICU healthcare workers during the COVID-19 pandemic and beyond. Heart Lung.

[80] Howell BAM (2021). Battling burnout at the frontlines of health care amid COVID-19. AACN Adv Crit Care.

[81] Santarone K, McKenney M, Elkbuli A (2020). Preserving mental health and resilience in frontline healthcare workers during COVID-19. Am J Emerg Med.

[82] Magner C, Greenberg N, Timmins F, O'Doherty V, Lyons B (2021). The psychological impact of COVID-19 on frontline healthcare workers 'From Heartbreak to Hope'. J Clin Nurs.

[83] Bagcchi S (2020). Stigma during the COVID-19 pandemic. Lancet Infect Dis.

[84] Le AB (2021). The health impacts of COVID-19-related racial discrimination of Asian Americans extend into the workplace. Am J Public Health.

[85] Rieckert A, Schuit E, Bleijenberg N, Ten Cate D, de Lange W, de Man-van Ginkel JM (2021). How can we build and maintain the resilience of our health care professionals during COVID-19? Recommendations based on a scoping review. BMJ Open.

[86] Walton M, Murray E, Christian MD (2020). Mental health care for medical staff and affiliated healthcare workers during the COVID-19 pandemic. Eur Heart J Acute Cardiovasc Care.

[87] Zeng Z, Wang X, Bi H, Li Y, Yue S, Gu S (2021). Factors that influence perceived organizational support for emotional labor of chinese medical personnel in Hubei. Front Psychol.

[88] Bullington G. RN perception of resources, communication, and leadership during covid-19 and the impact on RN satisfaction and turnover in a multi-hospital system. DNP Scholarly Projects. 2021;56. https://repository.belmont.edu/dnpscholarlyprojects/56.

[89] Nafar H, Tahmazi Aghdam E, Derakhshani N, Saniee N, Sharifian S, Goharinezhad S (2021). A systematic mapping review of factors associated with willingness to work under emergency condition. Hum Resour Health.

[90] Challinor JM, Alqudimat MR, Teixeira TOA, Oldenmenger WH (2020). Oncology nursing workforce: challenges, solutions, and future strategies. Lancet Oncol.

[91] Delaney RK, Locke A, Pershing ML, Geist C, Clouse E, Precourt Debbink M (2021). Experiences of a health system's faculty, staff, and trainees' career development, work culture, and childcare needs during the COVID-19 pandemic. JAMA Netw Open.

[92] Bellotti L, Zaniboni S, Balducci C, Grote G (2021). Rapid review on COVID-19, work-related aspects, and age differences. Int J Environ Res Public Health.

[93] Feng L, Yin R (2021). Social support and hope mediate the relationship between gratitude and depression among front-line medical staff during the pandemic of COVID-19. Front Psychol.

[94] Llop-Gironés A, Vračar A, Llop-Gironés G, Benach J, Angeli-Silva L, Jaimez L (2021). Employment and working conditions of nurses: where and how health inequalities have increased during the COVID-19 pandemic?. Hum Resour Health.

[95] Brugha R (2022). Doctor Retention in a COVID-World: an opportunity to reconfigure the health workforce, or "Plus ça change plus c'est la meme chose"? A response to the recent commentaries. Int J Health Policy Manag.

