# Supplementary material for: A global overview of healthcare workers’ turnover intention amid COVID-19 pandemic: a systematic review with future directions
Source: Hum Resour Health. 2022 Sep 24;20:70. doi: 10.1186/s12960-022-00764-7 (PMC9509627; doi:10.1186/s12960-022-00764-7)
Supplement: Supplementary file 1 — Additional file 1. Detailed search strategy for all databases. [file 12960_2022_764_MOESM1_ESM.docx]

**Detailed search strategy for all databases.**

**PubMed**

| #1 | “Physicians”[MeSH] OR “Nurses”[MeSH] OR “Physical Therapists”[MeSH] OR “Occupational Therapists”[MeSH] OR “Pharmacists”[MeSH] OR “Health Personnel”[MeSH] |
| --- | --- |
| #2 | “doctor”[tiab] OR “physician”[tiab] OR “nurs*”[tiab] OR “clinician”[tiab] OR “pharmacist”[tiab] OR “therapist”[tiab] OR “psychologist”[tiab] OR “allied health”[tiab] OR “healthcare professional”[tiab] OR “health professional”[tiab] OR “healthcare provider”[tiab] OR “healthcare worker”[tiab] OR “health worker”[tiab] |
| #3 | #1 OR #2 |
| #4 | “Personnel Turnover”[MeSH] |
| #5 | "turnover"[tiab] OR "attrition"[tiab] OR "retention"[tiab] OR "loyalty"[tiab] OR “intention to quit”[tiab] OR “intention to stay”[tiab] |
| #6 | #4 OR #5 |
| #7 | “COVID-19”[MeSH] |
| #8 | COVID-19[tiab] |
| #9 | #7 OR #8 |
| #10 | #3 AND #6 AND #9 |

**Embase**

| #1 | 'physician'/exp OR 'nurse'/exp OR 'health care personnel'/exp |
| --- | --- |
| #2 | doctor:ti,ab,kw OR physician:ti,ab,kw OR nurse:ti,ab,kw OR clinician:ti,ab,kw OR pharmacist:ti,ab,kw OR therapist:ti,ab,kw OR psychologist:ti,ab,kw OR 'allied health':ti,ab,kw OR 'healthcare professional':ti,ab,kw OR 'health professional':ti,ab,kw OR 'healthcare provider':ti,ab,kw OR 'healthcare worker':ti,ab,kw OR 'health worker':ti,ab,kw |
| #3 | turnover:ti,ab,kw OR attrition:ti,ab,kw OR retention:ti,ab,kw OR loyalty:ti,ab,kw OR 'intention to quit':ti,ab,kw OR 'intention to stay':ti,ab,kw |
| #4 | 'coronavirus disease 2019'/exp |
| #5 | 'covid-19':ti,ab,kw |
| #6 | #1 OR #2 |
| #7 | #4 OR #5 |
| #8 | #3 AND #6 AND #7 |

**CINAHL**

| S1 | (MH "Health Personnel") |
| --- | --- |
| S2 | TI ( doctor or physician or nurse or clinician or pharmacist or therapist or psychologist or "allied health" or "healthcare professional" or "health professional" or "healthcare provider" or "healthcare worker" or "health worker" ) OR AB ( doctor or physician or nurse or clinician or pharmacist or therapist or psychologist or "allied health" or "healthcare professional" or "health professional" or "healthcare provider" or "healthcare worker" or "health worker" ) |
| S3 | (MH "Personnel Turnover") |
| S4 | TI ( turnover or attrition or retention or loyalty or "intention to quit" or "intention to stay" ) OR AB ( turnover or attrition or retention or loyalty or "intention to quit" or "intention to stay" ) |
| S5 | (MH "COVID-19 Pandemic") |
| S6 | TI covid-19 OR AB covid-19 |
| S7 | S1 OR S2 |
| S8 | S3 OR S4 |
| S9 | S5 OR S6 |
| S10 | S7 AND S8 AND S9 |

**Scopus**

TITLE-ABS-KEY ( ( doctor OR physician OR nurse OR clinician OR pharmacist OR therapist OR psychologist OR "allied AND health" OR "healthcare AND professional" OR "health AND professional" OR "healthcare AND provider" OR "healthcare AND worker" OR "health AND worker" ) AND ( turnover OR attrition OR retention OR loyalty OR "intention AND to AND quit" OR "intention AND to AND stay" ) AND ( covid-19 ) )

**PsycINFO**

((doctor OR physician OR nurse OR clinician OR pharmacist OR therapist OR psychologist OR "allied health" OR "healthcare professional" OR "health professional" OR "healthcare provider" OR "healthcare worker" OR "health worker") and (turnover OR attrition OR retention OR loyalty OR "intention to quit" OR "intention to stay") and COVID-19).ab,ti.

**Web of Science**

In Title or Abstract: ( doctor OR physician OR nurse OR clinician OR pharmacist OR therapist OR psychologist OR "allied health" OR "healthcare professional" OR "health professional" OR "healthcare provider" OR "healthcare worker" OR "health worker" ) AND ( turnover OR attrition OR retention OR loyalty OR "intention to quit" OR "intention to stay" ) AND ( covid-19 )
